# Sirtuin-3-Mediated Cellular Metabolism Links Cardiovascular Remodeling with Hypertension

**DOI:** 10.3390/biology12050686

**Published:** 2023-05-06

**Authors:** Jing Gao, Weili Shen

**Affiliations:** Department of Cardiovascular Medicine, State Key Laboratory of Medical Genomics, Shanghai Key Laboratory of Hypertension, Shanghai Institute of Hypertension, Ruijin Hospital, Shanghai Jiao Tong University School of Medicine, Shanghai 200025, China

**Keywords:** SIRT3, metabolic reprogramming, cardiovascular remodeling, hypertension

## Abstract

**Simple Summary:**

Sirtuin-3 (SIRT3) performs a vital role in regulating metabolism, mitochondrial function, and oxidative stress. It has been connected to cardiovascular diseases, including hypertension. Studies indicate that hypertensive patients have reduced SIRT3 expression, leading to an upsurge in reactive oxygen species (ROS) levels and mitochondrial dysfunction. By deacetylating and activating antioxidants, SIRT3 helps regulate ROS levels. Moreover, SIRT3 activates enzymes responsible for the tricarboxylic acid (TCA) cycle and oxidative phosphorylation, thus enhancing mitochondrial respiration and ATP production, benefiting cardiovascular health.

**Abstract:**

Hypertension can cause structural and functional abnormalities in the cardiovascular system, which can be attributed to both hemodynamic and nonhemodynamic factors. These alterations are linked with metabolic changes and are induced by pathological stressors. Sirtuins are enzymes that act as stress sensors and regulate metabolic adaptation by deacetylating proteins. Among them, mitochondrial SIRT3 performs a crucial role in maintaining metabolic homeostasis. Evidence from experimental and clinical studies has shown that hypertension-induced decreases in SIRT3 activity can lead to cellular metabolism reprogramming and, subsequently, increased susceptibility to endothelial dysfunction, myocardial hypertrophy, myocardial fibrosis, and heart failure. This review presents recent research advances in SIRT3-mediated metabolic adaptation in hypertensive cardiovascular remodeling.

## 1. Introduction

Hypertension is responsible for approximately one-third of global deaths, making it the leading risk factor for cardiovascular diseases (CVDs) [1]. In the pathogenesis of chronic hypertension, myocardial and vascular function is initially intact and functional. However, over time, compensatory structural alterations occur that lead to variations in mass, size, geometry, and function [2,3]. Specific cardiovascular cells (such as endothelial cells, fibroblasts, smooth muscle cells, and cardiomyocytes) and immune cells can influence this cardiovascular remodeling in response to various metabolic, hemodynamic, and inflammatory stresses [4]. It is becoming increasingly evident that metabolic remodeling of cardiovascular cells promotes the remodeling of both the myocardium and arterial vasculature.

Sirtuins are a family of enzymes that rely on nicotinamide adenine dinucleotide (NAD^+^) for their deacetylating activity. There are seven sirtuin homologs (SIRT1-7) identified in mammals, which display a variety of subcellular localizations, enzymatic activities, and binding sites [5]. Among the sirtuins, SIRT3 is mainly localized to the mitochondrial matrix and performs a crucial role in regulating mitochondrial metabolism. SIRT3 protein is widespread in the cardiovascular system [6]. A growing body of evidence demonstrates that SIRT3 is essential for maintaining mitochondrial function by regulating acetylation modifications in hypertensive diseases, including endothelial dysfunction, cardiac hypertrophy, fibrosis, and heart failure [7]. This review highlights recent advances in the biological function and structure of SIRT3, and the metabolic mechanisms by which SIRT3 mediates cardiovascular remodeling in the context of hypertension.

## 2. SIRT3 Maintains Metabolic Homeostasis

Acetylation is a widespread posttranslational modification that coordinates metabolic flux, cell signaling, and cell differentiation. Mitochondria-related acetyl-CoA metabolism is critical for the acetylation of proteins and is a vital compartmentation point. Amongst sirtuins, SIRT3 exhibits the highest deacetylase activity and directly acts on various mitochondrial proteins involved in the modulation of mitochondrial energy metabolism, redox balance, and mitochondrial quality control [8].

### 2.1. Structural Properties of SIRT3

SIRT3 is an essential NAD^+^-dependent protein deacetylase localized in the mitochondria of mammalian cells, serving as a metabolic hub that controls the primary substrates responsible for mitochondrial metabolic homeostasis. SIRT3 displays a highly conserved enzymatic catalytic core region consisting of two major structural domains: a large Rossmann folding domain responsible for NAD^+^ binding and a small structural domain consisting of a helical bundle and a zinc-binding motif [9]. The cleft between the two domains can serve as a docking site for the acetylated peptide substrate. The cofactor binding pocket consists of three regions: (i) the adenine ribose moiety of NAD^+^, (ii) the nicotinamide ribose moiety, and (iii) the catalytic center located in the pocket’s deep region [9,10].

The human SIRT3 gene is located in the chromosome region 11p15.5. SIRT3 is initially synthesized in the cytoplasm, and its precursor is made up of 399 amino acids (44-kDa, inactive). In the nucleus, SIRT3 can deacetylate histone 3 (H3), H4, and Ku70. In the mitochondrial matrix, SIRT3 is cleaved by matrix processing peptidase (MPP) to generate the short active form of SIRT3, comprising 257 amino acids (28-kDa) that possess NAD-dependent deacetylase activity [11]. Many mitochondrial target proteins have been identified as SIRT3 substrates. In most cases, the deacetylation of lysine residues on the protein enhances their biological activity/function [12].

### 2.2. SIRT3 Modulates Fatty Acid Metabolism

Fatty acid (FA) metabolism comprises several steps, including FA synthesis, transport, and oxidation. FA metabolism dysfunction includes impaired carnitine and FA transport, CoA dehydrogenase deficiency, and ketone body production disorders [13]. SIRT3 modulates fatty acid metabolism by deacetylating key enzymes reversibly. Acetyl-CoA synthetase 2 (AceCS2) is an essential mitochondrial enzyme involved in the conversion of acetate to acetyl-CoA for the tricarboxylic acid (TCA) cycle, cholesterol, and fatty acid biosynthesis. SIRT3 deacetylates AceCS2 at Lys-642, and this activates the acetyl-CoA synthetase activity of AceCS2 [14]. On the other hand, the deletion of SIRT3 leads to the hyperacetylation of AceCS2, which inhibits its activity [14,15].

Carnitine palmitoyltransferase I (CPT1) is a crucial rate-limiting enzyme in FAO that regulates FAO in both healthy and disease states, promoting adaptation to the environment. CPT1 is predominant in the heart, skeletal muscle, and adipose tissue, and it mediates the entry of long-chain fatty acids into the mitochondria for β-oxidation reactions. SIRT3 knockout (KO) mice have been found to have more acetylated CPT1 than WT mice, indicating the important role of SIRT3 in regulating CPT1 activity [16]. FAO occurs in the mitochondrial matrix, and it undergoes a repetitive four-step reaction involving dehydrogenation, water addition, dehydrogenation, and thiolysis, each time releasing a molecule of acetyl-CoA. Acyl-CoA dehydrogenases (ACADs) catalyze the α and β dehydrogenation of acyl-CoA esters in fatty acids during the process. Currently, five types of ACADs have been identified and classified according to their substrate specificity: short-chain ACAD (SCAD), medium-chain ACAD (MCAD), long-chain ACAD (LCAD), very long-chain ACAD (VLCAD), and acyl-CoA dehydrogenase 9 (ACAD-9). Among them, long-chain acyl-CoA dehydrogenase (LCAD) has been extensively studied. Mass-spectrometry-based proteomics analysis of mitochondrial proteins shows that Lysine-42, Lys-318, and Lys-322 are targets for SIRT3 deacetylation [17]. In addition to lipogenesis and fatty acid oxidation, SIRT3 also enhances ketone body synthesis by deacetylating and activating hydroxyl methylglutaryl-CoA synthase 2 (HMGCS2), the rate-limiting step in β-hydroxybutyrate synthesis [18].

Fatty acid β-oxidation is a major source of mitochondrial oxidative phosphorylation in normal cardiac muscle. Since the heart has limited triacylglycerol stores, continuous intake of fatty acids ensures a fuel supply based on energy production. Therefore, FAO is the preferred pathway to obtain the energy required for efficient cardiac pumping, followed by the aerobic oxidation of glucose, lactate, and ketone bodies [19]. Mice lacking SIRT3 exhibit hyperacetylated LCAD, leading to a reduction in its enzymatic activity and impairing FAO in the heart [17]. Furthermore, a two-tiered proteomic approach identified other ACADs as SIRT3 substrates, including MCAD and ACAD9 [20].

Endothelial cells (ECs) not only form the inner wall of blood vessels but also rely on glucose glycolysis as their main source of energy. However, when glucose is not available, ECs can switch their metabolic flux towards increased FAO. Kalucka et al. [21] found that FAO in quiescent ECs is critical for maintaining redox homeostasis and EC functions. FAO is also essential for de novo dNTP synthesis in proliferative ECs [22]. CPT1α-mediated FAO regulates the proliferation of ECs in the stalk of the sprout [23,24]. A loss of CPT1α causes vascular sprouting defects both in vivo and in vitro [22]. Additionally, the pharmacological inhibition of CPT1α reduces FAO and enhances endothelial permeability [25,26].

### 2.3. SRIT3 Participates in Glucose Metabolism

Glucose metabolism is involved in several processes, such as glycogenesis, glycogenolysis, and gluconeogenesis. Members of the family of facilitative glucose transporters (GLUT-1 and GLUT-4) transport glucose into the heart. GLUT1 regulates insulin independence, while GLUT4 regulates insulin-stimulated glucose uptake. Inside the cell, hexokinase (HK) phosphorylates glucose to glucose-6-phosphate (G-6-P), which is then converted to pyruvate by glycolytic enzymes, including phosphofructokinase 1 (PFK1), PFK2, and pyruvate kinase (PK). Pyruvate can either be converted to lactate-by-lactate dehydrogenase (LDH) or transported through the mitochondrial pyruvate carrier (MPC) to the mitochondrial matrix. In the matrix, pyruvate is further oxidized to acetyl-CoA by the pyruvate dehydrogenase (PDH) complex for citric acid cycle metabolism. The adult heart exhibits metabolic flexibility, deriving a large portion of energy from fatty acids (70%), glucose, and lactose (20–30%) in its physiological state. Endothelial cells utilize glycolysis to generate ATP to promote sprouting, proliferation, and migration, making glucose homeostasis critical in the cardiovascular system. Previous studies suggested that SIRT3 is a critical regulator of glucose flux under conditions of nutrient overload, as systemic insulin sensitivity and insulin-stimulated muscle glucose uptake are significantly impaired. Lantier et al. [27] proposed that SIRT3 promotes the formation of the HKII-VDAC-adenine nucleotide transporter (ANT) complex on mitochondria, which enhances HKII activity, glucose uptake, and glucose oxidation. In the absence of SIRT3, HF-diet-fed SIRT3 KO mice exhibit reduced binding of HKII to mitochondria, associated with reduced formation of the VDAC-ANT complex.

SIRT3, a deacetylase, performs a crucial role in regulating the Warburg effect in mouse embryonic fibroblasts (MEFs). It mediates metabolic reprogramming in MEFs by destabilizing hypoxia-inducible factor-1α (HIF1α), which is a transcription factor that controls glycolytic gene expression. Loss of SIRT3 increases reactive oxygen species production, leading to the stabilization of HIF1α and upregulation of its target genes, including pyruvate dehydrogenase kinase 1 (PDK1), lactate dehydrogenase A (LDHA), and phosphoglycerate kinase (PGK1) [28]. Our experiments demonstrate that loss of SIRT3 induces hyperacetylation of endogenous autophagy-regulated gene 5 (ATG5), resulting in the inhibition of autophagosome maturation and increased expression of pyruvate kinase M2 (PKM2) dimer. Transgenic mice with EC-specific SIRT3 upregulation show reduced endothelial cell transition through improved glycolysis [29]. SIRT3 deacetylates and increases the activity of pyruvate dehydrogenase E1α (PDHA1), leading to a higher rate of pyruvate transformation into acetyl-CoA. This promotes glucose utilization and represses lactate production [30]. The discoveries suggest that SIRT3 represses glycolysis and inhibits lactate production, and further enhances glucose oxidation by activating the pyruvate dehydrogenase complex (PDC). It also targets enzymes involved in the tricarboxylic acid (TCA) cycle. Phosphofructokinase-2/fructose-2,6-bisphosphatase-3 (PFKFB3), a key regulator of glycolysis, has been reported to promote angiogenesis [31,32]. Conflicting data suggest that SIRT3 KO in ECs leads to defective angiogenesis with a reduction in the expression of PFKFB3, causing a significant decrease in basal glycolysis and glycolytic capacity compared to wild-type (WT) ECs [33,34]. The discrepancies in the effect of SIRT3 knockout on different cell types could be due to variations in experimental conditions, such as differences in cell culture conditions, animal models, and methods used to assess glycolysis. Additionally, compensatory mechanisms or alternative signaling pathways may be activated in response to SIRT3 knockout, which could influence the expression of PFKFB3 and other glycolytic enzymes. Hence, further research is required to fully comprehend the underlying mechanisms responsible for these inconsistencies.

### 2.4. SIRT3 Maintains Redox Homeostasis

Reactive oxygen species (ROS) consist of superoxide anions (O2•^−^), hydrogen peroxide (H_2_O_2_), and hydroxyl radicals (•OH^−^), which are tightly regulated by the antioxidant system. When there is an imbalance in ROS production and scavenging, oxidative stress occurs. Excess ROS can damage protein, lipid, and nucleic acid structures, leading to cytotoxic processes. The mitochondrial electron transport chain (ETC) is a major source of ROS. SIRT3 enhances mitochondria’s ROS coping ability in various ways. Previous studies have shown that isocitrate dehydrogenase (IDH), a key enzyme in the TCA cycle, is regulated by SIRT3-mediated deacetylation at lysine residues K75 and K241 [35,36]. Activated ICDH2 links calorie restriction-induced SIRT3 to preventing extensive age-related oxidative damage via the generation of NAD(P)H and aiding the conversion from oxidized to reduced glutathione [36]. SIRT3 can fully restore IDH2 activity to a maximum by deacetylating IDH2 at the Lys-413 site [37]. SIRT3 also binds and deacetylates glutamate dehydrogenase (GDH), an enzyme that converts glutamate to α-ketoglutarate, leading to increased NADPH production and decreased ROS levels. Regulating GDH by SIRT3 may be a way to control the entry of amino acids into the TCA cycle [38]. Apart from the TCA cycle, increasing evidence suggests that SIRT3 can bind directly to the NDUFA9 subunit of ETC complex I [39] and alpha subunits of ATP synthase (complex V), modulating oxidative phosphorylation activity through deacetylation [40]. NDUFA9 is important for cellular energy homeostasis and can indirectly contribute to redox homeostasis by reducing oxidative stress. Succinate dehydrogenase (SDH) is a unique enzyme involved in both the TCA cycle and oxidative phosphorylation. SDH is a part of complex II in the electron transport chain, and its regulation can also affect ROS production. SDHA subunits were also found to be SIRT3 substrates and hyperacetylated at lysine residues K179, K179, K485, K498, and K538 in SIRT3 KO mice [41]. Therefore, changes in the expression or activity of these enzymes can alter the balance of ROS production and antioxidant defense, ultimately affecting redox homeostasis.

Manganese superoxide dismutase (MnSOD) catalyzes the conversion of O_2_•^−^ to H_2_O_2_. H_2_O_2_ is further converted to H_2_O by catalase. SIRT3 has been shown to deacetylate lysine 122 of MnSOD and increase its activity, leading to the suppression of oxidative stress [42]. Peroxiredoxins (PRDXs) are another family of antioxidant enzymes that can efficiently scavenge peroxides such as H_2_O_2_, alkyl hydroperoxide, and peroxynitrite. A study by Wang et al. [43] revealed that SIRT3 activates PRDX3 by directly deacetylating K253. Furthermore, SIRT3-mediated deacetylation promotes the entry of transcription factor Forkhead box protein O3 (FoxO3a) into the nucleus, leading to increased transcription of antioxidant genes, including MnSOD, catalase, and PRDX3 [44]. In addition to enhancing the antioxidant system, SIRT3 also inhibits ROS generation. The main source of ROS is the mitochondrial ETC, and SIRT3 deficiency disrupts ETC, leading to electron leakage and ROS overproduction by increasing proton pump acetylation. Second, reducing factor NAD(P)H of glutathione reductase recycles oxidized glutathione back to its reduced form.

### 2.5. SIRT3 and Protein Metabolism

There is increasing evidence suggesting that SIRT3 performs a role in regulating the catabolism of amino acids. Glutamate dehydrogenase (GLUD1), responsible for converting glutamine to α-ketoglutarate, is one of the substrates of SIRT3 [45]. Additionally, SIRT3 performs a central role in the urea cycle (UC), as it deacetylates and activates the rate-limiting enzyme ornithine transaminase (OTC). Under fasted conditions, SIRT3 KO mice exhibit low OTC activity, leading to reduced amino acid catabolism and promoting ammonia toxicity, indicating a disturbance in nitrogen homeostasis [46]. SIRT3 has also been shown to deacetylate mitochondrial ribosomal protein L10 (MRPL10), the major acetylated protein in the mitochondrial ribosome. Ribosome-associated SIRT3 deacetylates MRPL10 in an NAD^+^-dependent manner. The MRPL10 from SIRT3 deficient mice, which is acetylated, exhibits greater efficiency in protein synthesis, leading to an elevation in the level of mitochondrially coded components of oxidative phosphorylation. The process of deacetylating MRPL10 may impede protein synthesis and lower the speed of oxidative phosphorylation by decreasing the expression of pivotal proteins involved in oxidative phosphorylation [47].

### 2.6. SIRT3 and Iron Metabolism

Iron is an essential element for diverse biological processes, and cellular iron homeostasis is tightly regulated by balancing circulation transport, uptake, export, and storage. Transferrin (TF)-bound iron in circulation is absorbed by binding to transferrin receptor (TFR1), and cells internalize the Iron-TF-TFR1 complex via receptor-mediated endocytosis. Ferric reductase six-transmembrane epithelial antigen of the prostate 3 (STEAP3) reduces free ferric iron (Fe^3+^) and facilitates its transportation across the endosomal membrane via divalent metal transporter 1 (DMT1). Cellular free iron is stored by forming a complex with ferritin, then exported to the circulation by the efflux protein ferroportin (FPN). An essential function of mitochondria is to regulate cellular iron balance by serving as a central hub for an iron-sulfur cluster (ISC) and heme production [48]. A previous study leveraging quantitative mass spectrometry has identified certain crucial iron homeostasis proteins, such as frataxin (FXN), aconitase 2 (ACO2), CDGSH iron-sulfur domain 3 (CISD3), glutaredoxin 5 (GLRX5), and thiosulfate sulfur transferase (TST), as potential targets of SIRT3-mediated deacetylation [49]. Hence, SIRT3 may participate in the regulatory network required for maintaining iron homeostasis. A deficiency of SIRT3 in renal tissue and pancreatic cancer cells leads to iron accumulation [50,51]. SIRT3 KO exacerbates Ang II-induced overexpression of iron overload-associated proteins in renal tissue, such as heme oxygenase (HO-1) and FPN [50]. Iron responsive elements (IREs) are short stem-loop structures in the 5′ and 3′ untranslated regions (UTRs) of mRNAs coding for iron-related proteins, the transcription efficiency of which is controlled by binding with iron regulatory proteins (IRPs). Loss of SIRT3 in pancreatic cancer cells increases IRP1 binding to IREs, and the expression of its target gene TFR1, thereby disrupting cellular iron homeostasis. The accumulation of excess labile iron generates ROS and highly reactive radicals, which can cause damage to lipids, proteins, and DNA within the body [51].

Ferroptosis is a form of programmed cell death characterized by NADPH oxidase activation, lipid peroxidation production, and ROS accumulation due to free iron overload [52]. Although SIRT3 deficiency induced iron accumulation in previous studies, it appeared to differently protect cells from ferroptosis [53,54]. In SIRT3-silent gallbladder cancer (GBC) cells, ferroptosis is inhibited through an AKT-dependent manner, and an increase in invasive activity and epithelial-mesenchymal (EMT) markers [53]. Additionally, in SIRT3-deficient cells, resistance to autophagy-dependent ferroptosis occurred by inhibiting the AMPK-mTOR signaling pathway and increasing glutathione peroxidase 4 (GPX4) expression [54]. Iron and SIRT3 exhibit a mutual interplay with each other. Iron overload downregulates SIRT3 protein expression, followed by hyperacetylation of SOD2, and also induces mitochondrion-derived superoxide anion-dependent autophagy, ultimately depressing bone marrow hematogenesis [55].

## 3. Role of SIRT3 in the Regulation of Vascular Cell Phenotypes

Clinical studies have shown a reduction in SIRT3 expression and activity in humans with systemic arterial hypertension. Vascular remodeling, which is associated with metabolic changes and involves multiple cell types, including endothelial cells, smooth muscle cells, and immune cells, is promoted by hypertension. This article sheds light on the significance of SIRT3 in regulating vascular cell phenotypes.

### 3.1. Angiogenesis

The process of angiogenesis heavily relies on glycolytic metabolism, where endothelial cells rely on high glycolytic rates to support sprouting, proliferation, and migration. Glycolysis accounts for up to 85% of total ATP production in endothelial cells [56,57]. Several studies have shown that angiogenic capability is impaired in SIRT3-deficient endothelial cells, which aggravates post-MI cardiac dysfunction [33]. Phosphofructokinase-2/fructose-2,6-bisphosphatase-3 (PFKFB3) is a crucial mediator of glycolysis and is known to boost angiogenic processes. He et al. [33,34] demonstrated that the decreased expression of PFKFB3 due to a loss of SIRT3 results in impaired glycolysis in endothelial cells, which contributes to coronary microvascular rarefaction and diastolic dysfunction in endothelial-specific SIRT3 KO mice. Our recent study revealed that endothelial cells exhibit defective autophagic flux and high levels of glycolysis when exposed to Ang II, along with a loss of mitochondrial SIRT3. SIRT3 ablation upregulates PKM2 dimer activity, promoting glycolysis [29]. Furthermore, hypertensive mice with endothelial-specific SIRT3 overexpression showed improved vascularity and cardiac function [58].

Apelin gene therapy has demonstrated significant improvements in the densities of capillaries and arterioles, and alleviating cardiomyopathy and myocardial infarction (MI) in the hearts of diabetic mice. Although apelin therapy has been shown to promote angiogenesis in animal models, SIRT3 KO mice exhibit resistance to the therapy, which suggests that SIRT3 is crucial for apelin-mediated angiogenesis. This is thought to be due to SIRT3 activation of the vascular endothelial growth factor (VEGF)/VEGFR2 and angiopoietin-1 (Ang-1)/Tie-2 signaling pathways [59]. Additionally, SIRT3 promotes angiogenesis through the activation of the Pink1/Parkin pathway, which increases rates of mitophagy in response to oxidative stress and inhibits the generation of ROS [58]. These findings partially reveal the regulatory role of SIRT3 in angiogenesis, but further detailed and specific studies are required.

### 3.2. Endothelial-Mesenchymal Transition (EndoMT)

EndoMT is a subset of epithelial-mesenchymal transition that contributes significantly to cardiovascular fibrosis by generating myofibroblasts. During EndoMT, endothelial cells (ECs) lose their endothelial markers, such as VE-cadherin and cluster of differentiation 31 (CD31), and acquire mesenchymal markers, such as α-smooth muscle actin (α-SMA) and fibroblast-specific protein 1 (FSP-1) [60], resulting in ECs exhibiting myofibroblast-like characteristics, such as contractile function, migratory phenotypes, and increased extracellular matrix production. Studies have shown that SIRT3 performs a crucial role in preventingEndoMT. For example, endothelial SIRT3 deficiency in obese mice worsened endothelial dysfunction and promoted transforming growth factor β-induced EndoMT [61,62]. Additionally, in a hypertension model, Lin et al. [63] found that the downregulation of SIRT3 expression resulted in increased acetylation of Foxo3a and inhibition of catalase expression. This SIRT3-Foxo3a-catalase pathway performs an essential role in the endothelial-to-mesenchymal transition and renal fibrosis. In ECs from diabetic kidneys, SIRT3 deficiency activates glycolysis by boosting the expression of glycolytic enzymes, such as HKII, PKM2 dimer, and pyruvate dehydrogenase kinase 4 (PDK4) via TGF-β/smad signaling-induced HIF1-α expression. Higher glycolysis levels lead to increased lactate production. These alterations result in EndoMT and can exacerbate kidney fibrosis both in vivo and in vitro [61,62].

**Figure 1:**
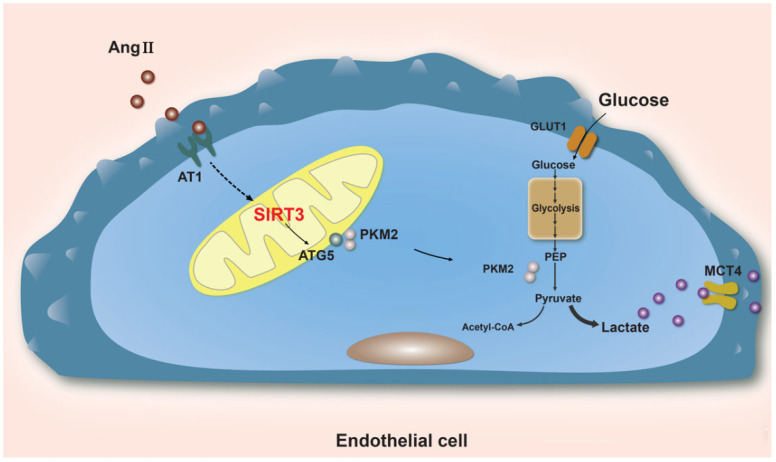
SIRT3 deficiency exacerbates the impairment of autophagy during the inducement of EndoMT by Ang II. Furthermore, autophagy suppression has been found to activate glycolysis. SIRT3 supports a high autophagy flux and downregulates glycolysis in ECs by deacetylating ATG5 and by degrading PKM2.

Our work indicates that metabolic changes rely on autophagic damage to drive endothelial cell phenotypic differentiation. In response to Ang II-induced EndoMT, SIRT3 deficiency exacerbates autophagy impairment while also activating glycolysis. SIRT3 deacetylation of ATG5 in endothelial cells is essential for maintaining high autophagic flux and suppressing glycolysis through PKM2 degradation. This contributes to the deceleration of EndoMT. Additionally, lactate produced by endothelial cells induces vascular smooth muscle cell (VSMC) differentiation into a synthetic phenotype. As a result, focusing on pharmacologically targeting endothelial cell metabolism could be an effective therapeutic approach for hypertensive vascular remodeling [29] (Figure 1).

### 3.3. Vascular Smooth Muscle Cell (VSMC) Phenotypes

Vascular smooth muscle cells (VSMCs) are a key component of arteries and perform a crucial role in vascular remodeling during hypertension. In physiological conditions, mature VSMCs display a contractile phenotype characterized by the expression of specific contractile proteins. However, in response to vascular injury, VSMCs undergo dedifferentiation into a synthetic phenotype, which is marked by increased proliferation, migration, and extracellular matrix production and is essential for the pathogenesis of vascular remodeling [64]. Growing evidence suggests that SIRT3-mediated changes in VSMC metabolism are associated with phenotypic switching. SIRT3 performs a vital role in blocking medial thickness by reducing VSMC proliferation and migration during vein graft remodeling and PDGF-BB-induced phenotype switching [65,66]. Our previous research has shown that Ang II-induced SIRT3 deficiency results in enhanced glycolytic flux and an increase in lactate release from ECs. Elevated lactate levels in the vascular microenvironment stimulate neighboring VSMCs, leading to a shift in phenotype towards the synthetic phenotype [29,67].

Moreover, Qiu and colleagues found that SIRT3 expression was significantly downregulated in the aortic tissues of Ang II-induced thoracic aortic dissection (TAD) mice, which increased the rupture rate of dissected vessels and the mortality of mice. The absence of SIRT3 caused an accumulation of ROS and increased apoptosis and the secretion of proinflammatory cytokines by VSMCs during TAD development [68]. SIRT3 is also involved in regulating VSMC calcification, and its upregulation induced by the deletion of soluble epoxide hydrolase (sEH) impairs VSMC calcification by preserving mitochondrial adenosine triphosphate (ATP) synthesis and morphology following high phosphorus (Pi) treatment [69]. Collectively, these findings suggest that reduced SIRT3 expression triggered by hypertension results in VSMC activation, leading to increased extracellular matrix secretion and vascular remodeling.

### 3.4. Proinflammatory Macrophages

Inflammation is a physiological response that performs a vital role in facilitating the healing process. However, the excessive inflammatory response induced by hypertension promotes reactive fibrosis and pathological remodeling. Chronic inflammation caused mainly by monocytes/macrophages is established as the primary cause of hypertensive vascular remodeling in many clinical trials [70,71]. Recent studies indicate that SIRT3 is a key regulator of the macrophage inflammatory response. Under Ang II infusion or endotoxin exposure, SIRT3 expression substantially decreases, leading to mitochondrial metabolic reprogramming, redox imbalance, and augmented secretion of inflammatory cytokines, such as TNF-α, IL-1β, and IL-6 in macrophages [72,73,74,75]. SIRT3 acts as a regulator of peroxisome proliferator-activated receptor alpha (PPARα) and AMP-activated protein kinase (AMPK), which are essential for maintaining mitochondrial homeostasis and activating autophagy activity. Autophagy performs a significant role in regulating inflammasome formation, initiation, and sustainment of inflammation. SIRT3-deficient macrophages exhibit a blockade in PPARα and transcription factor EB (TFEB) signaling pathways, thereby reducing autophagy and activating NLR family pyrin domain-containing 3 (NLRP3) inflammasomes [74,76,77]. SIRT3 deficiency-mediated pro-inflammatory effects in autophagy-induced NLRP3 inflammasome activation are well documented [73,74,76,77]. SIRT3 also modulates neuronal apoptosis inhibitory protein (NAIP)/NLR family caspase activation and recruitment domain-containing protein 4 (NLRC4) inflammasome, which induces IL-1β production and clearance of S. *typhimurium* through NLRC4 deacetylation in macrophages [78].

**Figure 2:**
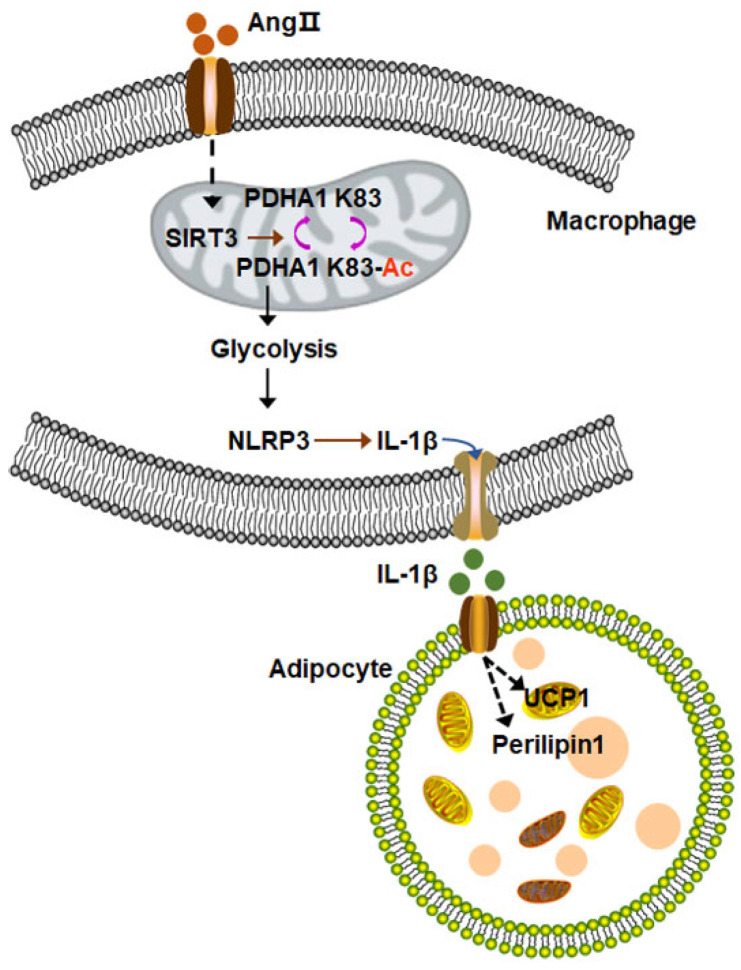
Loss of SIRT3 results in the increased acetylation of lysine 83 on PDHA1, which shifts the metabolic phenotype of macrophages from oxidative phosphorylation to glycolysis. Inflammatory macrophages induce the activation of NLRP3, leading to the release of IL-1β. Consequently, the levels of UCP1 and perilipin protein decrease while collagen IV deposition in PVAT increases.

Several studies have demonstrated a significant association between the inflammatory level of macrophages and glucose metabolism [79,80]. The loss of SIRT3 function results in increased acetylation levels of PDHA1 lysine 83 and induces a shift in the macrophage metabolic phenotype from oxidative phosphorylation to glycolysis. This metabolic reprogramming increases macrophagic inflammation by activating the NLRP3 inflammasome and promoting the release of IL-1β [72] (Figure 2).

In addition to glucose metabolism, the activation of macrophages and secretion of inflammatory cytokines also involves glutaminolysis. Cellular stress triggers the critical mechanism controlling SIRT3 activity, i.e., SUMOylation. SIRT3 is activated by IL-4 through SUMO-specific protease SENP1 translocation into mitochondria to deSUMOylate SIRT3 [81]. The enhanced expression of SIRT3 deacylates GLUD1, which catalyzes the conversion of glutamate into αKG. This accumulation of αKG leads to a shift in the metabolic pattern towards OXPHOS and a reduction in the trimethylation of histone H3K27 in the nucleus, thereby upregulating genes involved in M2 polarization. Activation of the SENP1-SIRT3-GLUD1 axis in macrophages inhibits the inflammatory response [82]. In summary, the above findings indicate that SIRT3 depletion triggers macrophage activation, proinflammatory cytokine secretion, and, consequently, chronic inflammation, which performs a crucial role in hypertensive vascular remodeling.

## 4. Effects of SIRT3 on Hypertensive Cardiac Remodeling

Metabolic disorders are a common feature of hypertensive heart disease, including hypertrophy, fibrosis, and heart failure. SIRT3, which regulates energy and redox metabolism and myocardial ATP levels, is abundantly expressed in the heart under conditions of high metabolic demand, thereby protecting the heart from metabolic disorders. While SIRT3 expression is variable under various physiological and pathological factors, its expression is positively regulated by exercise and caloric restriction. SIRT3 expression is increased during the early stages of cardiac hypertrophy induced by transverse aortic constriction (TAC), isoproterenol, or Ang II infusion. However, SIRT3 expression decreases upon progression to decompensated hypertrophy or heart failure, suggesting that it is responsive to changes in energy demand. Mitochondrial dysfunction and oxidative stress may be exaggerated from the adaptive oxidative capacity to meet ATP requirements to maladaptation in the failing heart. Consequently, SIRT3 is an attractive therapeutic target to improve metabolic abnormalities in hypertension.

### 4.1. Cardiac Hypertrophy

Chronic heart failure resulting from pressure overload is often accompanied by cardiac hypertrophy, an important pathological stage [1,2,3,4,5]. While initial cardiac hypertrophy is a compensatory process that supports myocardial contractility and cardiac function, sustained hypertrophy reduces myocardial compliance and cannot sufficiently meet the body’s blood pumping needs. As such, cardiac function declines over time and ultimately results in heart failure. SIRT3 performs a role in the development of various cardiovascular diseases, ranging from cardiac hypertrophy to dilated cardiomyopathy and heart failure. With age, SIRT3-deficient mice spontaneously develop cardiac hypertrophy, and they are more susceptible to hypertrophic responses induced by TAC, Ang II, or isoprenaline infusion than WT mice. Conversely, SIRT3 overexpression or activation via pharmacological agents can alleviate or even prevent cardiac hypertrophy under pressure overload or treatment with hypertrophic agonists [29,44].

Pathological cardiac stress leads to diminished fatty acid metabolism and accelerated glycolysis in hypertrophied hearts [83,84]. SIRT3 performs a role in inhibiting fatty acid synthesis by activating its inhibitor LKB1 through deacetylation, which subsequently enhances AMPK activity. In hypertrophic hearts, AMPK activation boosts the expression of fatty acid transporters CD36 and CPT1B. Meanwhile, AMPK activation further phosphorylates and inhibits ACC and MCD, synthesizing less malonyl-CoA, which is a negative regulator of fatty acid oxidation. Additionally, SIRT3 activates enzymes involved in fatty acid oxidation, including LCAD and TFP, through deacetylation. TFP catalyzes the β-oxidation of long-chain fatty acyl-CoAs using 2-enoyl-CoA hydratase (ECH), 3-hydroxyl-CoA dehydrogenase (HAD), and 3-ketothiolase (KT) activities consecutively [85].

Animal models of cardiac hypertrophy have demonstrated higher levels of glucose uptake, despite the abundance of glycolytic enzymes not being proportional to increased glycolysis. Reduced SIRT3 levels contribute to the promotion of glycolysis through several mechanisms. First, reduced SIRT3 levels lead to an increase in the acetylation of the PDC, which is responsible for linking glycolysis to the Krebs cycle. Inactivity of PDC results in mitochondrial dysfunction and prompts glycolysis [86]. Second, increased acetylation of the mitochondrial pyruvate carrier (MPC) reduces pyruvate import and PDH activity, despite no changes in protein abundance [87]. Third, SIRT3 enhances glucose metabolism in cardiomyocytes by indirectly upregulating the expression of PFKFB3 via apelin. Sundaresan et al. [44] have reported that SIRT3 can prevent cardiac hypertrophy by activating Foxo3a-dependent antioxidant enzymes, MnSOD, and catalase, which leads to the reduction in cellular levels of ROS. Reduced ROS levels inhibit Ras activation and downstream signaling via the MAPK/ERK and PI3K/Akt pathways, also inhibiting transcription factors, such as GATA binding protein 4 (GATA4) and T cell activation bioassay (NFAT) and translation factors, such as eukaryotic initiation factor 4E (elf4E) and S6 ribosomal protein (S6P), that are involved in the development of cardiac hypertrophy. Therefore, these findings imply that SIRT3 serves as an endogenous negative regulator of cardiac hypertrophy, protecting the heart by suppressing cellular ROS levels.

### 4.2. Cardiac Fibrosis

Cardiac fibroblasts (CFs) differentiate into myofibroblasts, which initiates myocardial fibrosis [88]. Myofibroblast conversion alters the metabolic state [89]. Normally, fibroblasts continuously modify the extracellular matrix (ECM) through production and degradation. However, when heart injury occurs (such as myocardial infarction or hypertension), proinflammatory factors are released, stimulating the fibroblasts to differentiate into highly proliferative and migratory myofibroblasts. These myofibroblasts help maintain the structural integrity of the injured heart [90], but over-deposition of the ECM can lead to pathological fibrosis and tissue dysfunction. Although specific and reliable marker proteins of myofibroblasts are not available, they can be identified through their formation of contractile bundles composed of actin and myosin, development of adhesion structures with substrate, and secretion of ECM [91]. Transforming growth factor-β (TGF-β) induces fibroblast differentiation to myofibroblasts, activating both the canonical downstream transcription factor Smad2/3 and the noncanonical mitogen-activated protein (MAP) kinase signaling pathway. TGF-β-Smad3 signaling is a major factor in cardiovascular fibrosis and cardiac hypertrophy that occurs due to fibroblasts stimulated by pressure overload [92].

SIRT3 KO mice exhibit cardiac fibrosis with aging and in response to hypertrophic agonists, inflammatory stimuli, or pressure overload. Studies show that SIRT3 deficiency spontaneously induces the trans-differentiation of fibroblasts to myofibroblasts, leading to fibrosis in the heart [93]. Treatment of fibroblasts with TGF-β results in decreased SIRT3 expression and an increased expression of fibrosis markers, such as smooth muscle α-actin (α-SMA), collagen-1, and fibronectin, which are indicators of fibroblast differentiation. Decreased SIRT3 expression leads to the acetylation of major antioxidative regulators, such as SOD2 and ICDH, and increased reactive oxygen species (ROS) production [94,95]. Furthermore, SIRT3 deficiency enhances SMAD3 expression and activates fibroblasts to transition into myofibroblasts, making SIRT3 KO mice more susceptible to pulmonary fibrosis (PF) than WT mice [95]. Excessive ROS oxidizes guanine residues to 7,8-dihydro-8-oxoguanine (8-oxodG), leading to DNA damage. OGG1, the DNA repair enzyme responsible for hydrolyzing 8-oxo-dG from DNA, is reduced in SIRT3-deficient fibroblasts, which leads to increased mtDNA damage and abnormal proliferation with the concomitant development of tissue fibrosis [44]. NaHS, an H_2_S donor, is a potential treatment for transverse aortic constriction (TAC)-induced myocardial fibrosis and Ang II-induced cardiac fibroblast proliferation by enhancing SIRT3 levels. It is necessary for NaHS-mediated attenuation of fibrosis marker expression in fibroblasts and amelioration of perivascular and interstitial collagen deposition by alleviating oxidative stress and improving mitochondrial respiration function and membrane potential [96]. Moreover, a loss of SIRT3 also causes a transformation from pericytes to fibroblasts by enhancing collagen I and TGF-β1 expression, making them susceptible to Ang II-induced renal fibrosis. Iron overload and the accumulation of ROS from NADPH oxidase are the primary mechanisms behind pericyte-to-fibroblast transition in kidneys deprived of SIRT3 [50]. These findings indicate that the loss of SIRT3 during hypertension progression may induce vascular remodeling by instigating the transformation of fibroblasts into myofibroblasts.

### 4.3. Heart Failure

Heart failure is characterized by impaired myocardial energy metabolism and reduced cardiac output. During the development of heart failure, reprogramming of myocardial metabolism occurs, shifting from fatty acid and glucose oxidation to glucose glycolysis, resulting in a reduction in overall mitochondrial oxidative metabolism and impaired ATP production [97]. In animal models of hypertensive heart failure, reduced levels of SIRT3 are observed, which are accompanied by mitochondrial protein lysine hyperacetylation [98]. Furthermore, SIRT3 KO mice exhibit reduced fatty acid oxidation, glucose oxidation, oxygen consumption, respiratory capacity, and ATP synthesis but increased glycolysis [99]. Zhang et al. [87] reported that SIRT3 expression is consistently lower in humans failing myocardium, and SIRT3 KO mice show higher levels of acetylation of PDH and ATP synthase, leading to decreased enzyme activity.

Angiotensin II is a major peptide of the renin-angiotensin system (RAS) and has emerged as a significant regulator of cardiac energy metabolism. Recent findings suggest that Ang II-induced reduction in glucose oxidation, leading to cardiac dysfunction, is jointly caused by insulin resistance and reduced PDH activity. Ang II reduces PDH activity through acetylation of the PDH complex and increased phosphorylation due to elevated levels of PDK4 [86]. Several other studies have suggested that Lys321 of the PDH E1α subunit [100], Lys259 and Lys480 of the ATP synthase β subunit [101], and Lys139 of ATP synthase [40] are potential regulatory targets for SIRT3. However, there is still controversy surrounding the correlation between acetylation and fatty acid β-oxidation-associated enzymatic activity. In contrast, Hirschey et al. [17] reported that a loss of SIRT3-induced LCAD hyperacetylation reduces its enzymatic activity, conflicting data have shown increased acetylation and activity of both LCAD and β-HAD in SIRT3 KO mice, particularly after HFD treatment [102]. The potential mechanisms underlying SIRT3-mediated metabolic alterations in cardiomyocytes are yet to be fully explored.

The interaction between endothelial cells (ECs) and cardiomyocytes is crucial for maintaining cardiomyocyte function. ECs support cardiomyocyte function by promoting angiogenesis. Impaired angiogenesis can lead to myocardial microvascular dysfunction, resulting in decreased oxygen and nutrient supply, which may contribute to the development of heart failure with preserved ejection fraction [103]. He et al. demonstrated that deleting SIRT3 in endothelial cells decreased PFKFB3-mediated glycolysis while increasing oxygen consumption rate and mitochondrial oxidative stress. Ablation of SIRT3 in ECs increases oxygen consumption and limits oxygen supply to cardiomyocytes, which may exacerbate cardiac hypoxia and apoptosis [34,104]. The loss of endothelial SIRT3-induced reprogramming of glycolytic metabolism may contribute to the development of coronary microvascular rarefaction and heart failure.

## 5. Conclusions

Mitochondria are essential organelles that perform a crucial role in metabolic homeostasis and energy supply. One of the key regulators of mitochondrial metabolic function is SIRT3, which achieves this through reversible protein lysine deacetylation. Here, we examine the potential roles of SIRT3 in cardiovascular remodeling in hypertension. It has been debated whether SIRT3 has anti-hypertensive effects. Studies conducted on hypertensive mice infused with high-dose Ang II have shown that the gain or loss of SIRT3 function does not seem to have an impact on blood pressure [58,72]. However, Dikalova et al. [105] found that SIRT3 does exhibit anti-hypertensive effects in mice infused with a smaller dose of Ang II. It appears that the hypotensive effects of SIRT3 may depend on the level of blood pressure. As such, this paper did not focus on exploring the potential hypotensive effects of SIRT3.

Recent pharmacological research has identified agents that activate SIRT3 and confer cardiovascular protection. For instance, Losartan, an angiotensin II type I receptor agonist, has been shown to protect against ischemia-reperfusion injuries through SIRT3 activation [106]. Metformin mitigates VSMC premature senescence by AMPK activation induced by SIRT3 [107]. Ecklonia cava extract (ECE) decreases hypertension-related vascular calcification through SOD2 SIRT3-deacetylation [108]. Administration of alpha-linolenic acid (ALA) restores endothelial cell autophagic flux and mitochondrial redox stress via increased SIRT3 activity, which improves endothelial cell dysfunction and attenuates experimental hypertension. These findings suggest that interventions targeting SIRT3 activity may have potential clinical applications in treating cardiovascular disease.

## Data Availability

No new data were created or analyzed in this study.
